# Qualitative and quantitative study of fetal posterior fossa during the first trimester in a Chinese population

**DOI:** 10.1186/s12884-022-05086-z

**Published:** 2022-10-10

**Authors:** Li Feng, Lijuan Sun, Jingjing Wang, Congxin Sun, Lijuan Lu, Zhikun Zhang, Yu Hu, Qingqing Wu

**Affiliations:** 1 Beijing Obstetrics and Gynecology Hospital, Capital Medical University, Beijing, P. R. China; 2 Beijing Maternal and Child Health Care Hospital, Beijing, P. R. China; 3 Shijiazhuang Obstetrics and Gynecology Hospital, Shijiazhuang, P. R. China; 4 Kunming Maternity and Child Care Hospital, Kunming, P. R. China; 5 Tianjin Central Hospital of Gynecology Obstetrics, Tianjin, P. R. China; 6 Department Ultrasound, Beijing Obstetrics and Gynecology Hospital, Capital Medical University, No. 251 Yaojiayuan Road, Chaoyang District, 100026 Beijing , P. R. China

**Keywords:** Fetal posterior fossa, First trimester, Parameters

## Abstract

**Background:**

To establish the normal reference ranges for parameters related to the fetal posterior fossa in the first trimester (11 ~ 13+6 weeks of gestation) and to analyze the relationship between them and crown-rump length (CRL) among the Chinese population.

**Methods:**

Singleton pregnancies of 11 ~ 13^+6^ weeks (CRL:45 ~ 84 mm) with both parents from China were randomly selected from January 2021 to November 2021. The related parameters of the posterior fossa including cisterna magna (CM), intracranial translucency (IT), brain stem (BS), brain stem to the occipital bone (BSOB), and brain stem/brain stem to occipital bone (BS/BSOB) were evaluated and measured in nuchal translucency (NT) mid-sagittal section clearly by an experienced sonographer (operator 1). To assess the reproducibility of the measurements, we randomly selected 50 pregnant women. According to the blind method, operators 1 and 2 respectively screened and measured relevant parameters. In addition, operator 1 examined and measured relevant parameters again 2 h after the first.

**Results:**

This study included 1663 fetuses. All fetuses can clearly show the three spaces of the fetal posterior fossa. The ICCs (95% CI) of intra-operator reproducibility of CM, IT, BS, BSOB, BS/BSOB were 0.981 (0.952 ~ 0.991, *P* < 0.001), 0.929 (0.866 ~ 0.961, *P* < 0.001), 0.970 (0.946 ~ 0.983, *P* < 0.001), 0.991 (0.974 ~ 0.996, *P* < 0.001), 0.939 (0.892 ~ 0.965, *P* < 0.001), respectively; The ICCs (95% CI) of inter-operator reproducibility 0.926 (0.860 ~ 0.960, *P* < 0.001), 0.810 (-0.083 ~ 0.940, *P* < 0.001), 0.820 (0.645 ~ 0.904, *P* < 0.001), 0.804 (0.656 ~ 0.888, *P* < 0.001), 0.772 (0.599 ~ 0.871, *P* < 0.001), respectively. There was a linear correlation between CRL and the parameters related to the posterior fossa (CM, IT, BS, BSOB, BS/BSOB). CM (mm) = -1.698 + 0.532 × CRL (cm) (*r* = 0.829, *P* < 0.001); IT (mm) = 0.701 + 0.179 × CRL (cm) (*r* = 0.548, *P* < 0.001); BS (mm) = 0.403 + 0.349 × CRL (cm) (*r* = 0.716, *P* < 0.001); BSOB (mm) = -0.277 + 0.719 × CRL (cm) (*r* = 0.829, *P* < 0.001); BS/BSOB = 0.747—0.021 × CRL (cm) (*r* = 0.196, *P* < 0.001).

**Conclusions:**

Qualitative and quantitative assessment of the fetal posterior fossa structure was feasible in the first trimester. We constructed the normal reference ranges of CM, IT, BS, BSOB, and BS/BSOB. Furthermore, CM, IT, BS, and BSOB were positively correlated with CRL, but BS/BSOB was negatively correlated with CRL.

## Background

Central nervous system malformations (CNSM) are the most common anomalies after fetal heart defects [1]. In congenital fetal anomalies, CNSM accounts for 40 to 50 percent and 75 percent of the causes of fetal death during pregnancy [2]. At present, the diagnosis of posterior fossa malformations such as Dandy-Walker malformation (DWM), Blake’s pouch cyst (BPC), Open spina bifida (OSB), and Joubert syndrome were mostly concentrated in the second and third trimester, which is mainly since the cerebellar hemispheres and vermis are not yet well developed in the first trimester. Nowadays, with the improvement of ultrasound instrument resolution and sonographer’s expertise, many guidelines and expert consensus have recommended that assessment of the posterior fossa anatomy be included in the routine first trimester screening. Most scholars in the world had found that abnormalities of some relevant indirect markers (CM, IT, BS, BSOB, BS/BSOB) in the posterior fossa during the first trimester could predict the possible existence of the above malformations [3–5], providing the best time for early detection, diagnosis, and intervention, which to some extent reduces the physiological and psychological trauma to pregnant women and improves the birth quality of our population. However, there is no standard for the normal reference ranges of relevant parameters of the posterior fossa in China.

The purpose of our study was, firstly, to qualitatively assess whether the three spaces of the fetal posterior fossa was normal or not, and secondly, to establish the normal reference ranges for parameters related to the fetal posterior fossa during the first trimester and analyze the relationship between them and CRL.

## Methods

### Study setting, duration and sample size

This study collected pregnant women who underwent fetal NT examination at 11 ~ 13 + 6 weeks in the ultrasound department of Beijing Obstetrics and Gynecology Hospital, Capital Medical University. Beijing Maternal and Child Health Care Hospital from January 2021 to November 2021. We explained the specific details of our study to these pregnant women. For pregnant women who volunteered to participate, we signed informed consent. Based on the expected date of childbirth, we followed up through the PACS and HIS systems to determine the pregnancy outcome. At the same time, we also inquired about the situation of the newborn in detail at the 42-day postpartum review. If the information was incomplete, we followed up by telephone. As of May 2022, information on all pregnant women had been collected.

### Study inclusion and exclusion criteria

The inclusion criteria were as follows: (1) singleton pregnancy; (2) fetal ultrasound estimated gestational age matched with gestational menstrual age; (3) CRL between 45 and 84 mm; (4) fetuses without significant abnormalities on NT scanning.

The exclusion criteria were as follows: (1) twin pregnancy; (2) fetal ultrasound estimated gestational age was not matched with gestational menstrual age; (3) pregnant women with uncertain or unknown gestational ages; (4) fetuses or newborns with structural or soft index abnormalities as demonstrated by subsequent ultrasound screening and 42-day postpartum follow-up; (5) miscarriage or stillbirth.

### Study instrument and procedure

Using Samsung WS80A color Doppler ultrasound diagnostic equipment, the abdominal ultrasound probe was used for scanning, and the probe models were CA1-7A and CV1-8A, with probe frequencies of 1 ~ 7MHZ and 1 ~ 8MHZ, respectively.

According to the guidelines issued by Fetal Medicine Foundation (FMF) [6], the International Society of Ultrasound in Obstetrics and Gynecology (ISUOG) [7], the American Institute of Ultrasound in Medicine(AIUM) [8], the requirements for prenatal ultrasound screening in the first trimester were as follows.

**Fig. 1:**
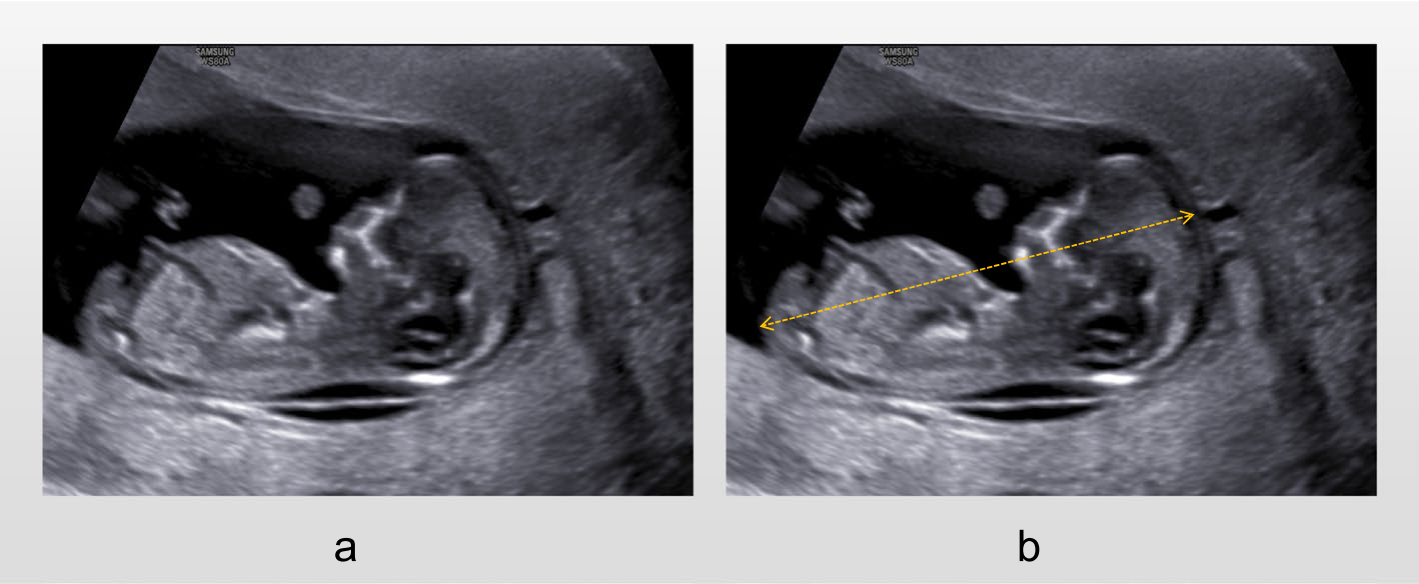
CRL standard section (**a**) and measurement standards (**b**)

**Fig. 2:**
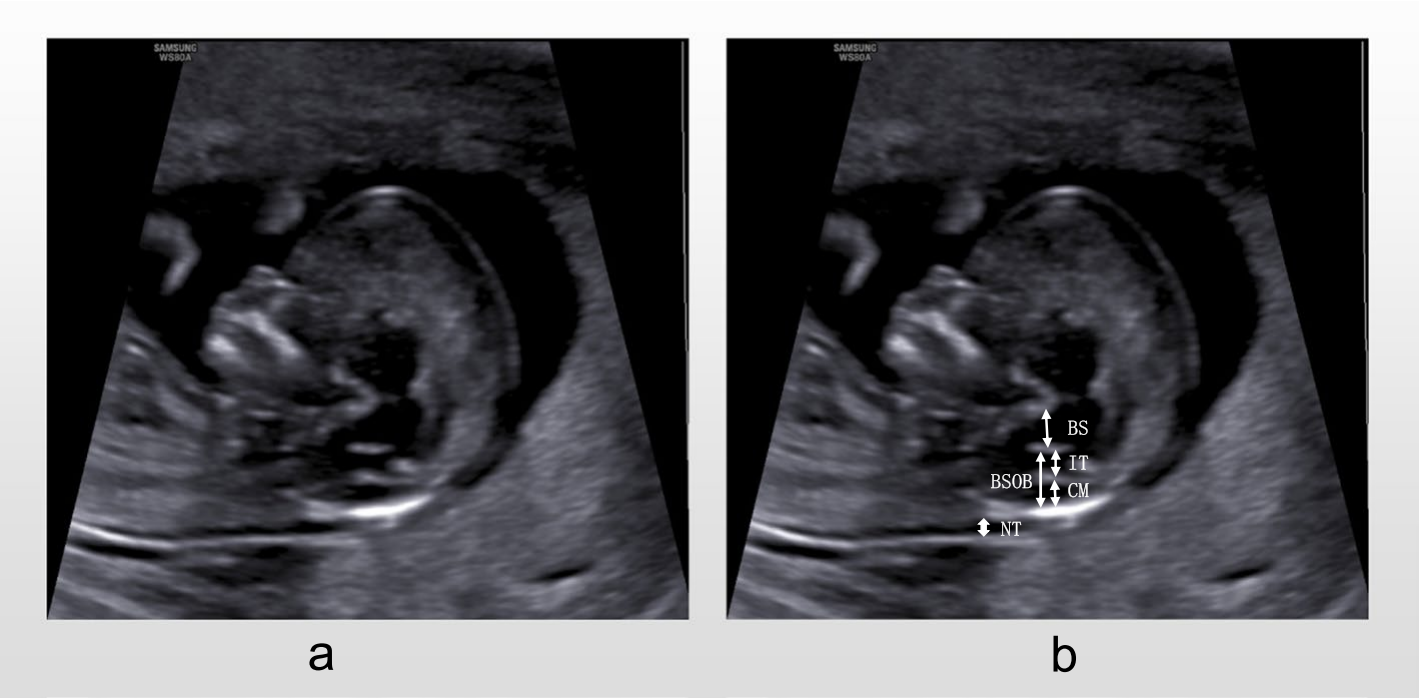
NT standard section (**a**) and measurement standards of related parameters (**b**)

① Fetal mid-sagittal view: the fetus was in a neutral position, and the image of interest was enlarged to more than 2/3 of the ultrasound image area, clearly showing the outer edges of the skin of the sacrococcygeal and top of the head, the skin of the anterior nasal area and the tip of the nose, the nasal bone, the palate, the mandible, the three intracranial structures, the complete dorsal skin, and the spine (Fig. 1a).
② Fetal NT section: the fetus was in a neutral position, showing only the head, neck, and upper chest. The image of interest was enlarged to more than 2/3 of the ultrasound image area, clearly showing the fetal anterior nasal skin and nasal tip, the nasal bone, the palate, the mandible, the three intracranial structures, the spine, the soft tissues, skin and translucent area of the back of the head and neck (Fig. 2a).

### Qualitative and quantitative assessment of the fetal posterior fossa

Three spaces and four lines could be seen between the sphenoid bone and the occipital bone in the posterior fossa of the normal fetus. From front to back, the three spaces are BS, IT, and CM, and the four lines are the posterior border of the sphenoid bone, the posterior border of the brain stem and the anterior border of the fourth ventricle, the choroid plexus of the fourth ventricle(4VCP) and the anterior border of cisterna magna, and the anterior border of the occipital bone respectively. Meanwhile, fetal CRL, NT, vertical distance of CM, IT, BS, BSOB, and BS/BSOB needed to be measured. All parameters were measured by an experienced sonographer (operator 1). The distance from the outer edge of the most prominent part of the top of the fetal head to the outer edge of the sacrococcygeal skin was measured in the fetal mid-sagittal view as CRL (Fig. 1b); the NT value was measured in the fetal NT section by placing the inner border of the callipers at the widest point of the translucent area on the back of the head and neck. BS distance was measured between the posterior border of the sphenoid bone and the brain stem; IT was defined as the vertical distance between the anterior border of the fourth ventricle and the 4VCP; the distance between the cisterna magna anteriorly and the occipital bone anteriorly was CM; the BSOB vertical distance referred to the anterior border of the fourth ventricle and the occipital bone (Fig. 2b). To assess the reproducibility of the measurements, we randomly selected 50 pregnant women. According to the blind method, operators 1 and 2 respectively screened and measured relevant parameters during the examination of pregnant women. In addition, operator 1 examined and measured relevant parameters again 2 h after the first. All parameters are measured three times and the average value is taken.

### Statistical analysis

SPSS 26.0 and MedCalc statistical software were used, and the measurement data were first tested by the Kolmogorov–Smirnov normality test, and those that obeyed Gaussian distribution were expressed as mean ± standard deviation, and those that did not obey Gaussian distributions were expressed as median (first quartile, third quartile). ICC and Bland–Altman plots were used to analyze the intra-operator and inter-operator agreement, and ICC > 0.75 was considered good. The normal reference ranges of correlation parameters with a normal distribution were expressed as mean ± 1.96 standard deviations, and those not obeying normal distribution were expressed as a percentile. Correlations between CRL and each parameter were analyzed by Person correlation analysis and linear correlation, and regression equations were derived.

### Ethical consideration

The study was approved by our Ethics Committee of Beijing Obstetrics and Gynecology Hospital, Capital Medical University (Ethics approval number:2019-KY-106–01), and all pregnant women were informed in detail and signed informed consent to voluntarily participate in this study.

## Results

A total of 1885 pregnant women were initially included in this study. 29 cases were excluded due to excessive maternal fat and uncooperative fetal posture, and 193 cases were excluded due to fetal structural abnormalities, miscarriage, and stillbirth found on follow-up ultrasound screening or in labour. Thus, a total of 1663 cases were involved in the final analysis. It is worth noting that the three spaces of the fetal posterior fossa can be clearly displayed in all subjects. All pregnant women with CRL (4.5–8.4 cm) were divided into 4 subgroups according to CRL interval 1 cm group in the first trimester. The basic characteristics of the study population was shown in Table 1. Since all parameters did not conform to the normal distribution, they were expressed as median (first quartile, third quartile) in Table 1.

**Table 1:** The basic characteristics of the study population

CRL(cm)	Number	Age(years)	CM(mm)	IT(mm)	BS(mm)	BSOB(mm)	BS/BSOB
4.5 ~ 5.4	80	31.0 (28.0,34.0)	1.1 (1.0,1.2)	1.7 (1.5,1.8)	2.2 (2.0,2.4)	3.5 (3.3,3.7)	0.61 (0.57,0.69)
5.5 ~ 6.4	749	31.0 (29.0,33.0)	1.4 (1.3,1.6)	1.8 (1.7,1.9)	2.5 (2.4,2.7)	4.0 (3.8,4.3)	0.62 (0.57,0.68)
6.5 ~ 7.4	728	31.5 (29.0,34.0)	2.0 (1.8,2.1)	1.9 (1.8,2.0)	2.8 (2.6,2.9)	4.6 (4.4,4.9)	0.60 (0.57,0.64)
7.5 ~ 8.4	106	32.0 (29.0,35.0)	2.4 (2.1,2.6)	2.1 (2.0,2.3)	3.1 (2.9,3.2)	5.3 (5.0,5.6)	0.58 (0.55,0.62)

### Agreement between intra‐operator and inter‐operator measurements

**Fig. 3:**
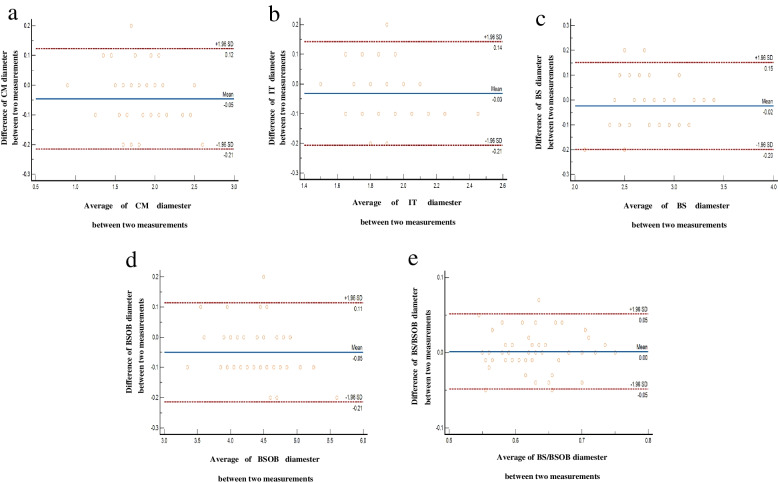
Intra-operator agreement in the measurements of CM (**a**), IT (**b**), BS (**c**), BSOB (**d**), BS/BSOB (**e**); solid line: the mean of the difference of; the paired measurement dotted line: 95%LOA of the difference

**Fig. 4:**
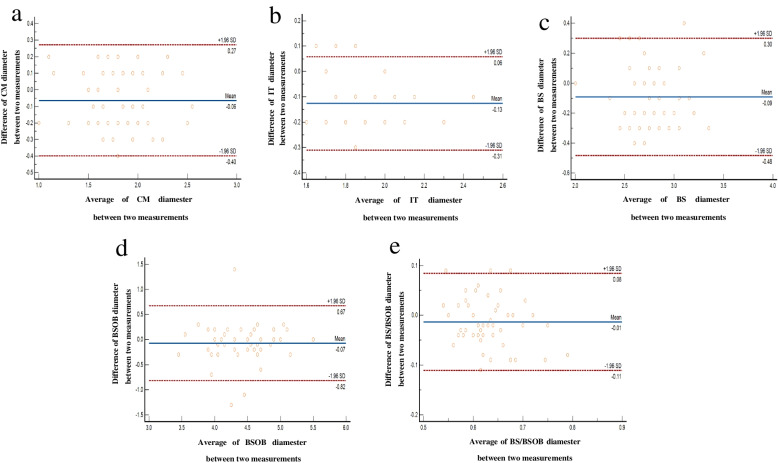
Inter-operator agreement in the measurements of CM (**a**), IT (**b**), BS (**c**), BSOB (**d**), BS/BSOB (**e**); solid line: the mean of the difference of; the paired measurement dotted line: 95%LOA of the difference

The ICCs (95% CI) of intra-operator reproducibility of CM, IT, BS, BSOB, BS/BSOB were 0.981 (0.952 ~ 0.991, *P* < 0.001), 0.929 (0.866 ~ 0.961, *P* < 0.001), 0.970 (0.946 ~ 0.983, *P* < 0.001), 0.991 (0.974 ~ 0.996, *P* < 0.001), 0.939 (0.892 ~ 0.965, *P* < 0.001), respectively; The ICCs (95% CI) of inter-operator reproducibility were 0.926 (0.860 ~ 0.960, *P* < 0.001), 0.810 (-0.083 ~ 0.940, *P* < 0.001), 0.820 (0.645 ~ 0.904, *P* < 0.001), 0.804 (0.656 ~ 0.888, *P* < 0.001), 0.772 (0.599 ~ 0.871, *P* < 0.001), respectively; the ICCs were all greater than 0.75. Meanwhile the good consistency between intra-operator and inter-operator could be more visually derived from the Bland–Altman plots (Figs. 3 and 4).

### Correlation between various parameters of the posterior fossa and CRL

**Fig. 5:**
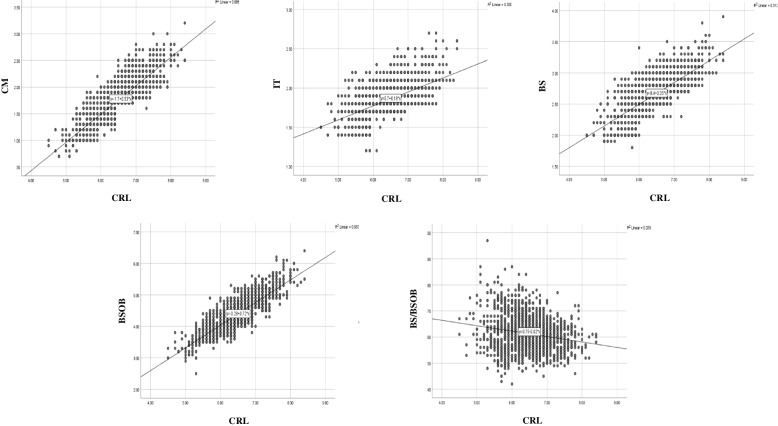
Correlation between various parameters of the posterior fossa and CRL

CM, IT, BS, and BSOB were positively correlated with CRL, but BS/BSOB was negatively correlated with CRL. CM (mm) = -1.698 + 0.532 × CRL (cm) (*r* = 0.829, *P* < 0.001); IT (mm) = 0.701 + 0.179 × CRL (cm) (*r* = 0.548, *P* < 0.001); BS (mm) = 0.403 + 0.349 × CRL (cm) (*r* = 0.716, *P* < 0.001); BSOB (mm) = -0.277 + 0.719 × CRL (cm) (*r* = 0.829, *P* < 0.001); BS/BSOB = 0.747—0.021 × CRL (cm) (*r* = 0.196, *P* < 0.001). The scatter plots of the relationship between each parameter and CRL were shown in Fig. 5.

### Normal reference ranges of various parameters of the posterior fossa

The normal reference ranges of CM, IT, BS, BSOB, and BS/BSOB were shown in Table 2.

**Table 2:** The normal reference ranges of CM, IT, BS, BSOB, BS/BSOB for different CRL intervals

CRL	parameter	percentile						
**(cm)**	**(mm)**	**5**	**10**	**25**	**50**	**75**	**90**	**95**
4.5–5.4	CM	0.7	0.8	1.0	1.1	1.2	1.4	1.5
	IT	1.4	1.4	1.5	1.7	1.8	1.9	2.1
	BS	1.9	2.0	2.0	2.2	2.4	2.6	2.7
	BSOB	3.0	3.1	3.3	3.5	3.7	3.9	3.9
	BS/BSOB	0.53	0.54	0.57	0.61	0.69	0.77	0.81
5.5–6.4	CM	1.1	1.2	1.3	1.4	1.6	1.8	1.9
	IT	1.5	1.6	1.7	1.8	1.9	2.0	2.1
	BS	2.0	2.1	2.4	2.5	2.7	2.8	2.9
	BSOB	3.6	3.7	3.8	4.0	4.3	4.5	4.7
	BS/BSOB	0.51	0.53	0.57	0.62	0.68	0.72	0.75
6.4–7.4	CM	1.7	1.7	1.8	2.0	2.1	2.3	2.4
	IT	1.7	1.7	1.8	1.9	2.0	2.2	2.2
	BS	2.5	2.5	2.6	2.8	2.9	3.1	3.1
	BSOB	4.1	4.2	4.4	4.6	4.9	5.2	5.3
	BS/BSOB	0.52	0.53	0.57	0.60	0.64	0.67	0.70
7.5–8.4	CM	2.0	2.0	2.1	2.4	2.6	2.7	2.8
	IT	1.8	1.8	2.0	2.1	2.3	2.4	2.6
	BS	2.8	2.8	2.9	3.1	3.2	3.4	3.5
	BSOB	4.9	4.9	5.0	5.3	5.6	5.8	6.1
	BS/BSOB	0.51	0.52	0.55	0.58	0.62	0.64	0.67

## Discussion

Fetal posterior fossa anomalies in the first trimester are closely associated with fetal structural malformations and chromosomal abnormalities [9–12]. AIUM recently published recommendations for fetal anatomy screening in the first trimester, which includes the evaluation of the fetal posterior fossa structure. At 11–13^+6^ weeks, the NT mid-sagittal section can clearly show the relevant structure of the fetal posterior fossa, and it significantly improves the evaluation of the fetal posterior fossa structure with increasing gestational age [13, 14]. This viewpoint was also confirmed in our study. In recent years, more and more scholars have begun to investigate fetal CNS malformations by qualitative and quantitative assessment of fetal posterior fossa anatomy in the first trimester [15–17].

Initially, posterior fossa structures were first observed to screen for OSB [18, 19]. In the prospective multicenter Berlin IT-study, Chih-Kang CF et al. [20] included 11 cases of OSB and five markers of CM, IT, BS, BSOB, and BS/BSOB. They found that OSB was screened for in all cases, with eight cases detected at the first examination and the remaining three suspicious cases detected several weeks later, and demonstrated that the IT and CM of incorporated parameters could be used to predict OSB. Engels et al. [4] in their review of the literature also proposed that posterior fossa markers can be used to predict OSB. The main signs were the absence of one of the three spaces, IT showing poorly, CM showing poorly or below the 5th percentile, BS above the 95th percentile, BSOB below the fifth percentile, and BS/BSOB values above the 95th percentile. The sensitivity of CM, IT, BS, BSOB, and BS/BSOB were 50% to 73%, 50%, 96.7%, 86.7%, and 100%, respectively, and the specificity of IT is as high as 99%.

Subsequently, with everyone’s further discussion and understanding of the structure of the posterior fossa, it was later also gradually applied to predict cystic deformities of the posterior fossa (including DWM, BPC, mega cisterna magna, Joubert’s syndrome, etc.) [17, 21, 22]. In Italy, P. Volpe et al. included 32 fetuses with widened BSOB and/or failure to visualize the 4VPC in the first trimester, 18 cases were excluded because autopsy results could not be obtained, and the remaining 14 cases were finally confirmed to have 4 cases of DWM, 8 cases of BPC, and 2 cases of normal. Garcia-Rodriguez R et al. declared that chromosomal abnormalities were found 13 of 14 fetuses in which a cystic posterior fossa was detected in the first trimester, and the IT and CM of fetuses with cystic posterior fossa are markedly different from normal fetuses. In the study of Martinez-Ten P et al. [23], 28 fetus with non-visible IT from Spain and Chile were included in the first trimester, 12 of which had central nervous system abnormalities (including OSB = 6, DWM = 2, BPC = 2, mega cisterna magna = 1, cephalocele = 1), and 20 of 28 cases associated with aneuploidy chromosomes. CNS or chromosomal abnormalities were present in all 21 cases included by P. Volpe et al. [24] in which one of the three spaces of the posterior fossa was absent.

In summary, the diagnosis of fetal posterior fossa abnormalities was mainly based on qualitative and quantitative markers (CM, IT, BS, BSOB and BS/BSOB). Any abnormal diagnosis was derived from the definition of normal. The standard for qualitative diagnosis of the normal posterior fossa structure was to clearly display “three spaces and four lines”, but there is no unified standard for quantitative evaluation of related posterior fossa parameters in China [25–27]. Eric Ozdemir M et al. [28] demonstrated that IT value, BSOB value, and BS/BSOB ratio were identified as ultrasound variables for predicting posterior fossa anomalies with a sensitivity of 100%, 100%, and 100%; and specificity of 95.9%, 94.7%, and 98.5%, respectively in Istanbul. The largest study on the establishment of a normal reference ranges for parameters related to the posterior fossa was from abroad and included 15,526 pregnant women. However, the reference ranges for BSOB and BS/BSOB differed significantly from the our study, and the possible explanation for this was ethnic diversity [20]. In addition, there were few related studies in China, and their commonality was that the sample size was small and all parameters obeyed the normal distribution, which was inconsistent with the multicenter Berlin study and our study. Therefore, based on a relatively large sample date, we established the normal reference ranges of parameters related to the fetal posterior fossa in the first trimester in line with my country’s national conditions, which provided an important reference for early detection of structural abnormalities in the posterior fossa.

Applying the normal reference ranges of BSOB values obtained from our study to the BSOB measurements listed in the 17 cases of suspected anomalies of the cystic posterior fossa included in the study of P. Volpe et al. [29] revealed that 16 cases were greater than the 95th percentile, which was generally consistent with the 15 cases greater than the 95th percentile obtained in that study. In our study, CM, IT, BS, and BSOB were found to be positively correlated with CRL, and BS/BSOB was negatively correlated with CRL, which was consistent with the results of most investigators [13, 28, 29]. This result suggested that it was reasonable and necessary to establish a normal reference range based on the CRL grouping. Most studies did not group the normal reference values of parameters related to the posterior fossa in early pregnancy or divided them into three groups by gestational age only. We chose to divide them into four subgroups at 1 cm intervals of CRL to give more accurate normal reference ranges corresponding to different gestational age. In addition, our intra- and inter-operator consistency assessment results for all parameters of posterior fossa showed that the ICC of all parameters was greater than 0.75, proving its good reproducibility. However, the shortcomings of this research were the single-center study and the small sample date in the CRL intervals of 4.5–5.4 cm and 7.5–8.4 cm, further multicenter study is warranted.

## Conclusions

It’s feasible to assess fetal posterior fossa anatomy qualitatively and quantitatively in the first trimester. Intra- and inter-operator reproducibility for related parameters (CM, IT, BS, BSOB, BS/BSOB distance) of posterior fossa were good. We established the normal reference ranges of parameters related to the fetal posterior fossa in the first trimester in line with my country’s national conditions. In our study, CM, IT, BS, and BSOB were found to be positively correlated with CRL, and BS/BSOB was negatively correlated with CRL. It’s crucial to evaluate the fetal posterior fossa structure during the first trimester.

## Data Availability

The datasets used and/or analyzed during the current study are available from the corresponding author on reasonable request.

## Abbreviations

CRL: Crown-rump length
CM: Cisterna magna
IT: Intracranial translucency
BS: Brain stem
BSOB: Brain stem to the occipital bone
BS/BSOB: Brain stem diameter/brain stem to occipital bone distance
NT: Nuchal translucency
ICC: Intraclass correlation coefficient
CNSM: Central nervous system malformations
DWM: Dandy-Walker malformation
BPC: Blake’s pouch cyst
OSB: Open spina bifida
FMF: Fetal Medicine Foundation
ISUOG: International Society of Ultrasound in Obstetrics and Gynecology
AIUM: American Institute of Ultrasound in Medicine
4VCP: Choroid plexus of the fourth ventricle
